# Tanshinone IIA combined with CsA inhibit myocardial cell apoptosis induced by renal ischemia-reperfusion injury in obese rats

**DOI:** 10.1186/s12906-021-03270-w

**Published:** 2021-03-22

**Authors:** He Tai, Xiao-lin Jiang, Zhi-ming Lan, Yue Li, Liang Kong, Si-cheng Yao, Nan Song, Mei-jun Lv, Jin Wu, Ping Yang, Xuan-si Xiao, Guan-lin Yang, Jin-song Kuang, Lian-qun Jia

**Affiliations:** 1 Key Laboratory of Ministry of Education for Traditional Chinese Medicine Viscera-State Theory and Applications, Liaoning University of Traditional Chinese Medicine, Shenyang, China; 2 Department of Nephrology, The fourth of Affiliated Hospital of Guangzhou University of Traditional Chinese Medicine (Shenzhen Traditional Chinese Medicine Hospital), Guangzhou University of Traditional Chinese Medicine, Shenzhen, China; 3 Department of Medical laboratory, The fourth of Affiliated Hospital, Guangzhou University of Traditional Chinese Medicine (Shenzhen Traditional Chinese Medicine Hospital), Guangzhou University of Traditional Chinese Medicine, Shenzhen, China; 4 School of Pharmacy, Liaoning University of Traditional Chinese Medicine, Dalian, China; 5 Department of Cardiovascular Medicine, The Affiliated Hospital of Liaoning Traditional Chinese Medicine, Shenyang, China; 6 Department of Endocrinology and Metabolic, Shenyang the Fourth Hospital of People, Shenyang, China

**Keywords:** Renal ischemia-reperfusion, Obesity, Mitochondrial dysfunction, Acute myocardial injury, Tanshinone IIA, Cyclosporine a, Apoptosis, PI3K/Akt/bad pathway

## Abstract

**Background:**

Acute myocardial injury (AMI), which is induced by renal ischemia-reperfusion (IR), is a significant cause of acute kidney injury (AKI)-related associated death. Obesity increases the severity and frequency of AMI and AKI. Tanshinone IIA (TIIA) combined with cyclosporine A (CsA) pretreatment was used to alleviate myocardial cell apoptosis induced by renal IR, and to determine whether TIIA combined with CsA would attenuate myocardial cell apoptosis by modulating mitochondrial function through the PI3K/Akt/Bad pathway in obese rats.

**Methods:**

Male rates were fed a high fat diet for 8 weeks to generate obesity. AKI was induced by 30 min of kidney ischemia followed 24 h of reperfusion. Obese rats were given TIIA (10 mg/kg·d) for 2 weeks and CsA (5 mg/kg) 30 min before renal IR. After 24 h of reperfusion, the rats were anaesthetized, the blood were fetched from the abdominal aorta and kidney were fetched from abdominal cavity, then related indicators were examined.

**Results:**

TIIA combined with CsA can alleviate the pathohistological injury and apoptosis induced by renal IR in myocardial cells. TIIA combined with CsA improved cardiac function after renal ischemia (30 min)-reperfusion (24 h) in obese rats. At the same time, TIIA combined with CsA improved mitochondrial function. Abnormal function of mitochondria was supported by decreases in respiration controlling rate (RCR), intracellular adenosine triphosphate (ATP), oxygen consumption rate, and mitochondrial membrane potential (MMP), and increases in mitochondrial reactive oxygen species (ROS), opening of the mitochondrial permeability transition pore (mPTP), mitochondrial DNA damage, and mitochondrial respiratory chain complex enzymes. The injury of mitochondrial dynamic function was assessed by decrease in dynamin-related protein 1 (Drp1), and increases in mitofusin1/2 (Mfn1/2), and mitochondrial biogenesis injury was assessed by decreases in PPARγ coactivator-1-α (PGC-1), nucleo respiratory factor1 (Nrf1), and transcription factor A of mitochondrial (TFam).

**Conclusion:**

We used isolated mitochondria from rat myocardial tissues to demonstrate that myocardial mitochondrial dysfunction occurred along with renal IR to induce myocardial cell apoptosis; obesity aggravated apoptosis. TIIA combined with CsA attenuated myocardial cell apoptosis by modulating mitochondrial function through the PI3K/Akt/Bad pathway in obese rats.

## Background

Acute kidney injury (AKI) is a very severe, even life-threatening complication of critically ill patients [1]. Renal ischemia-reperfusion (IR) is the main cause for AKI [2]. Because hyperlipidaemia, diabetes and hyperuricaemia are closely related to obesity and can induce insulin resistance and hypertension, they are considered as chronic hyperinflammatory [3], which can increase the severity and incidence of renal diseases [4]. Ischemia and AKI rarely occur in the kidneys alone, as they are often combined with multiple organ dysfunction. Patients with renal insufficiency have a high-risk of triggering cardiovascular diseases, representing 45% of the cause of death in patients undergoing hemodialysis [5]. The mechanism of acute myocardial injury (AMI) induced by renal IR is indeterminate, including kidney dysfunction which results in a hyper-inflammatory state and volume overload [6]. Renal IR can lead to impactful changes in heart morphology combined with increased microvasculature [7]. The study has demonstrated that the IR mechanism is closely related with mitochondrial dysfunction in heart [8]. However, no study has explored mitochondrial dysfunction in myocardial cells induced by renal IR.

Reducing the inflammatory response and resisting oxidant stress are the main biological activities of tanshinone IIA (TIIA), the main active ingredient in *Salvia miltiorrhiza* Bge [9]. In addition, TIIA plays a protective role in myocardial ischemia [10]. The study has demonstrated that TIIA represses opening of the mitochondrial permeability transition pore (mPTP), which is cardioprotective [11]. Cyclosporine A (CsA) inhibits opening of the mPTP by binding to cyclophilin D (CyP-D) to minimize IR damage [12]. Injecting CsA before ischemia protects renal function [13]. However, few studies have investigated the myocardial protective properties of TIIA and CsA by repressing opening of the mPTP.

Cell survival is associated with the phosphoinositide-3 kinase (PI3K) pathway, which relies on Akt kinase phosphorylation and activation followed by the proapoptotic Blc-2 family protein Bad phosphorylation and inhibition. PI3K plays an important role in growth factor signal transduction. Under various cytokines and the activation of physiochemical factors, PI3K generates myoinositol as a second messenger. Akt also plays crucial roles in many biological processes, such as the cell cycle, cell metabolism, apoptosis, and cell growth [14]. Mitochondrial-mediated apoptosis is inhibited through the PI3K/Akt/Bad signaling pathway [15]. However, few studies have investigated the myocardial protective roles of TIIA and CsA by modulating mitochondrial function through the PI3K/Akt/Bad pathway.

The current research focused on assessing an AKI method (induced by IR) leading to dysfunction of myocardial mitochondria, using a combination of TIIA and CsA to identify new therapeutic targets.

## Methods

### Experimental animals and ethical statement

We used male Sprague–Dawley rats (weight 180–220 g and 8 weeks old) in our experiments. The animals were acquired from Liaoning Changsheng Biotechnology Co., Ltd. (Production License: SCXK (Liao) 2015–0001). The rats were kept in cages under controlled conditions of 20 ± 3 °C and 45–65% humidity, and with a 12 h light/dark cycle (lights on at 06:00 h), and fed water and a pelleted diet ad libitum. Animal experimental design and husbandry procedures were approved by the Ethical Committee of Animal Handling (2019019) at Liaoning University of Traditional Chinese Medicine and complied with the guidelines of the Use and Care of laboratory animals published by US National Institutes of Health. We did our best to decrease the number of rats and suffering during the study.

### Chemicals and reagents

TIIA (Sulfotanshinone Sodium Injection, 10 mg each) was produced at the Shanghai NO. 1 Biochemical Pharmaceutical Co., Ltd. (Shanghai, China). TIIA was dissolved in deionized water to obtain a stock solution (5 mg/ml). CsA (20 mg each) was produced at Beijing Solarbio Science & Technology Co., Ltd. (Beijing, China) and was dissolved in 0.1% dimethylsulfoxide to a stock solution (2.5 mg/ml), which was further diluted to achieve the ideal concentration. All of the above stock solutions were used in a timely manner.

### Animal grouping and methods of drug dosing

A total of 120 rats were randomly (random number table method) divided into six groups, including the sham operation group, the IR group, the IR (obese) group, the TIIA group, the CsA group, and the TIIA+ CsA group. Each group included 20 rats. All rats were fed a general maintenance feed for 2 weeks to adapt to the environment. Then, the sham and IR groups continued to eat the general maintenance feed for 8 weeks, whereas the other four groups were fed a high-fat diet (HFD) for 8 weeks. Rats in the sham group, the IR, and IR (obese) groups were given deionized water. The TIIA group was given an intraperitoneal injection of TIIA (10 mg/kg·d) for 2 weeks before renal IR; the CsA group was given an intraperitoneal injection of CsA (5 mg/kg) 30 min before renal IR; and the TIIA + CsA group was given an intraperitoneal injection of TIIA (10 mg/kg·d) for 2 weeks before renal IR+ intraperitoneal injection CsA (5 mg/kg) 30 min before renal IR. The components of the HFD included 25% total fat, 18% protein, 44% carbohydrate, 13% fiber, and 11% unsaturated fat, ash, and other ingredients [16]. Rats that gained 30% of their body weight were chosen for further study [17].

### Surgical procedure

The rats were anaesthetized with thiopental sodium (120 mg/kg) by intraperitoneal injection, and pinching of the tail and paw was used to evaluate the anesthetic effect. The abdomen was opened to expose the right kidney, and the renal pedicle was separated to expose the renal vessels. A 3–0 silk suture was used to ligate the renal vessel, and the right kidney was removed. The left kidney was exposed and the renal vessels were separated. An arterial clamp was used to clamp off the left renal artery for 30 min to build ischemia. After 30 min of ischemia, the arterial clamp was removed for 24 h of reperfusion. The left kidney was observed for 15 min to ensure normal blood reperfusion, which was shown by a red color [18]. The incision was closed with 3–0 silk sutures. The rats were placed on a heating pad to maintain 37 °C body temperature throughout the experimental procedure. Sham-operated rats received the same surgical procedure without using an arterial clamp to clamp off the left renal artery [19].

### Serum myocardial enzyme spectrum and inflammatory factor analyses

Arterial blood samples were used for the myocardial enzyme spectrum analysis. A 0.5 mL aliquot of arterial blood was drawn from the abdominal aorta (at the end of the reperfusion period), and creatine kinase isoenzymes-muscle/brain (CK-MB), cardiac troponin I (cTNI), tumor necrosis factor-α (TNF-α), and interleukin-1β (IL-1β) were measured with kits (KHB, Shanghai, China).

### Cardiac function analysis

**Fig. 1:**
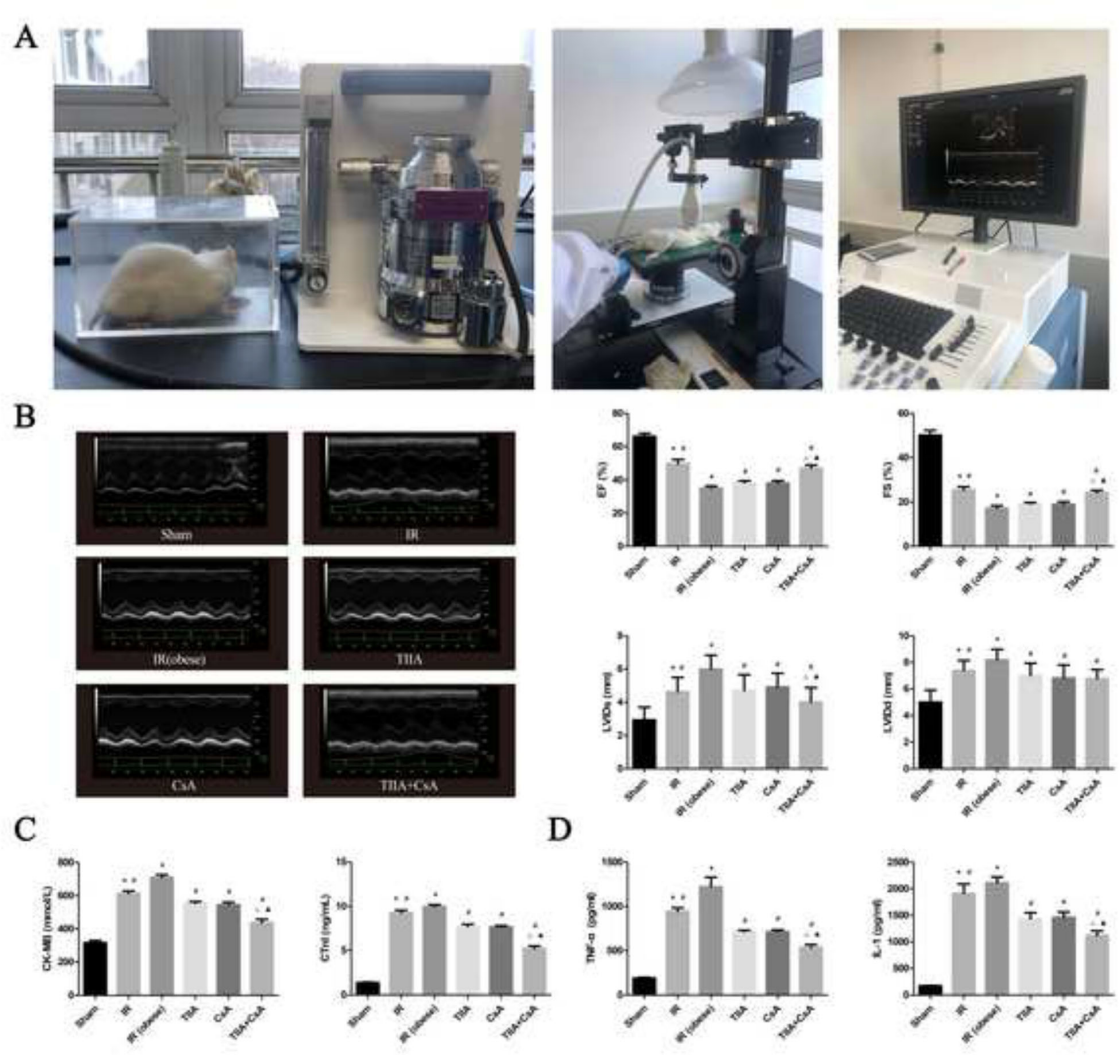
Tanshinone IIA (TIIA) + cyclosporine A (CsA) improved the cardiac function, myocardial enzyme, and inflammatory factor after renal ischemia-reperfusion (IR). Rats were pre-treated with TIIA alone or in combination with CsA followed by removing the right kidney and clamping of the left renal artery for 30 min and reperfusion for 24 h. Sham rats were used as control. Cardiac function (**b**), myocardial enzyme (**c**), and inflammatory factor (**d**) were evaluated under different groups. Data are shown as mean ± SD. *p < 0.05 versus sham group, ^#^*p* < 0.05 versus IR (obese) group, ^△^*p* < 0.05 versus TIIA group, ^▲^*p* < 0.05 versus CsA group

Noninvasive transthoracic echocardiography (vevo2100; Visualsonics, Toronto, ONT, Canada) was used to evaluate the function and morphology of the left ventricle in anesthetized animals (Matrx VIP 3000) (Fig. 1a). Animals were anesthetized with 1–2% isofurane in 100% oxygen, placed on a heating pad (temperature was preserved at 37 °C). This method consisted of two-dimensional mode. Long-axis and mid-papillary level shortaxis B-mode images were used to measure left ventricular (LV). Left ventricular end-systolic internal diameter (LVIDs) and left ventricular end-diastolic internal diameter (LVIDd), End-systole was defned at minimal, while end-diastole was defned at maximal LVID. The left ventricular ejection fraction (EF) and left ventricular fractional shortening (FS) were recorded, FS was counted as FS = (LVIDd- LVIDs)/ LVIDd× 100% [20].

### Histological assessment of the myocardium by hematoxylin and eosin (HE) staining

Myocardial tissues were cut into 5-μm thick sections and stained with HE after paraffin embedding as described previously [21]. We used paraformaldehyde (4%) to immerse the myocardial tissues for 24 h and then transferred the tissues to ethanol (70%). The myocardial tissues were observed under light microscopy [22]. The degree of injury in myocardial tissues was scored on a scale from 0 to 4 [23]: 0, normal myocardial tissues; 1 (mild), interstitial edema and localized necrosis; 2 (moderate), widespread myocardial cell swelling and necrosis; 3 (severe), necrosis with contraction bands, neutrophil infiltration, and compressed capillaries; and 4 (highly severe), diffuse necrosis with contraction bands, neutrophil infiltration, and compressed capillaries and hemorrhaging.

### Electron microscopy to observe mitochondria

Myocardial tissues were obtained immediately after anesthesia and cut into small pieces (1 mm^3^). The specimens were fixed in glutaraldehyde (2%) at 4 °C, washed with pH 7.4 PBS (0.1 mol/L), fixed with osmium tetroxide (1%), and stained with aqueous uranyl acetate (1%). Place the pecimens at 70 °C for about 48 h with capsules embedding medium. The sections were stained with uranyl acetate and alkaline lead citrate for observation with an electron microscope (H-7650; Hitachi, Tokyo, Japan).

### Apoptosis assessment of the myocardial tissue using TUNEL

The terminal deoxynucleotidyl transferase-mediated dUTP nick end-labeling (TUNEL) assay was used to detect apoptosis with an In Situ Cell Death Detection Kit, POD (Roche, Mannheim, Germany). We used 5-μm thick sections for TUNEL staining [21]. The sections were incubated for 15 min in Protease K (10 μg/mL) after deparaffinization and rehydration. A fresh TUNEL reaction mixture was added to the samples, which were incubated (37 °C) for 60 min in the dark. After washing, 0.1 μg/mL 4′,6-diamidino-2-phenylindole (DAPI) (Beyotime) was used to stain the cell nuclei. The samples were analyzed in a drop of phosphate buffered saline (PBS) under a fluorescence microscope (Canon, Tokyo, Japan). We observed eight random visual fields per animal to determine the number of TUNEL-positive cells in a high-power field.

### Caspase-3/9 activity

Caspase-3/9 activity were detected in myocardial tissues by the fluorescent caspase-specific substrate AcDEVD-7-pNA (Solarbio). A 10 mg portion of myocardial proteins was added to reaction buffer and incubated at 37 °C for 2 h. Enzyme-catalyzed release was quantified by a fluorimeter at 405 nm.

### Preparation of the mitochondrial suspension

As previously reported in liver [24], the animals were sacrificed by cervical dislocation, and the heart was quickly removed and placed in an ice-cold pH 7.4 buffer (1 mM EDTA, 250 mM sucrose, and 10 mM Tris-HCl). After trimming, the myocardial tissues (50–100 mg) were homogenized in an isolation buffer. The entire isolation process was performed at 4 °C. The supernatant was collected after centrifugation in 700×g for 10 min, and the supernatant was centrifuged again at 7000×g for 10 min. The resulting supernatant was discarded, and the mitochondrial pellet was resuspended in 5 ml isolated buffer followed by centrifuging twice at 7000×g for 10 min. A clean mitochondrial solution was acquired and preserved in mitochondrial preservation solution (20 mM sucrose, 10 mM KH_2_PO_4_, 2 mM MgCl_2_, 100 mM KCl, 5 mM HEPES, and 1 mM EDTA) to generate a 5 mg/mL mitochondrial protein suspension, which was placed on ice for immediate use. The bicinchoninic acid (BCA) reagent box (Beyotime) was used to measure the protein concentration of the mitochondrial suspension (100–1000 μg/ml). The mitochondrial suspension was used to measure the mitochondrial membrane potential (MMP), ATP synthesis, opening of the mitochondrial permeability transition pore (mPTP), reactive oxygen species (ROS), and the respiratory control rate (RCR).

### Measurement of the MMP

A mitochondrial membrane potential assay kit (Beyotime) was used to detect the MMP. The prepared JC-1 working solution was diluted five times with JC-1 Dyeing Buffer (1X); a 0.9 ml aliquot of the diluted JC-1 working solution was added to 0.1 ml of the purified mitochondria (protein content 100–1000 μg/ml). We used a fluoroenzyme label to detect MMP; 1 ml of the mixture was added to the fluoroenzyme-labeled assay with the emission wavelength set to 530 nm and excitation wavelength set to 490 nm, and the red/green value was calculated.

### Opening of the mPTP

Opening of the mPTP was measured by detecting absorbance at 540 nm of mitochondria exposed to 250 μM CaCl_2_; “-”: treatment without Ca^+^; “+”: treatment with Ca^+^. The Purified Mitochondrial Membrane Pore Channel Colorimetric Assay kit (GENMED, Shanghai, China) was used according to the manufacturer’s protocol. CaCl_2_ (200 μmol/L) was used to induce opening of the mPTP. The optical density (OD) value from 0 to 10 min at 520 nm was read from an ultra-micro microporous plate spectrophotometer (Biotek, Winooski, VT, USA). A decrease in the OD value reflects opening of the mPTP. The OD value recorded at the onset of the experiment (0 min) represents minimum optical density (min OD); the OD value recorded at the end of the experiment (10 min) represents maximum optical density (min OD). Min/max OD is negatively associated with the extent of MPTP opening [24, 25].

### Measurement of ROS

ROS were detected using the multi-mode microplate reader and the 2′,7′-dichlorodihydrofluorescein diacetate fluorescent probe (DCFH-DA).

### Measurement of oxygen consumption rate and RCR

Mitochondrial oxygen consumption rate was detected using a Clark-type electrode connected to a thermostatic water bath and calibrated with using air-saturated medium (contain 432 nmol O/ml at 32 °C).

RCR (ratio of state III to state IV) is an index used to assess oxidative phosphorylation, respiratory chain function, and integrity of isolated mitochondria. The RCR was evaluated using an Oxytherm Clark-type oxygen electrode (OXYT1/ED; Hansatech Instruments, Norfolk, UK). Mitochondria (60 μg isolated from myocardiu) were placed in the oxytherm chamber containing respiration buffer (20 mM HEPES, 125 mM KCl, 0.1% BSA, 2.5 mM KH_2_PO_4_, and 2 mM MgCl_2_, pH 7.2), and stirred at 37 °C. The slope of the response of mitochondria to consecutive administrations of respiration substrates was defined as the rate of oxygen consumption for each respiratory state, as reported previously [26].

### Measurement of damaged mtDNA

Damaged mitochondrial DNA (mtDNA) was assessed through the ratio of long and short fragments using the real-time quantitative polymerase chain reaction (RT-qPCR). DNA was isolated by extracting total DNA using the Genomic-tip 20/G kit (Qiagen, Valencia, CA, USA) according to the manufacturer’s protocol. The PCR products and purified DNA were quantified fluorometrically using the Picogreen ds DNA reagent (Invitrogen, Milan, Italy). RT-qPCR was performed on the DNA extracts as reported previously using the following modifications [27]. PCR amplification was conducted using the Ranger DNA Polymerase with appropriate premixes (Bioline Ltd., London, UK). The two primer pairs (mtDNA long fragment and short fragment) (General Biosystems, Anhui, China) are shown in Table 1. The mtDNA long fragment was amplified using the standard thermocycler program, including initial denaturation at 94 °C (1 min), 94 °C (15 s) for 18 cycles, 65 °C (12 min), and a final extension at 72 °C (10 min). The short mtDNA fragment was amplified using the same program except the extension temperature was 60 °C.

**Table 1:** Sequence of primers for RT-PCR and long PCR

Target Gene	Primer Sequence	Size (bp)	Tm (°C)
Mfn1	Forward: 5′-GGGAAGACCAAATCGACAGA-3′	152	57
	Reverse: 5′-CAAAACAGACAGGCGACAAA-3′		57
Mfn2	Forward: 5′-GAGAGGCGATTTGAGGAGTG-3′	165	58
	Reverse: 5′-CTCTTCCCGCATTTCAAGAC-3′		56
Drp1	Forward: 5′-GCCCGTGGATGATAAAAGTG-3′	215	56
	Reverse: 5′-TGGCGGTCAAGATGTCAATA-3′		56
PGC-1α	Forward: 5′-GGACGAATACCGCAGAGAGT-3′	201	59
	Reverse: 5′-CCATCATCCCGCAGATTTAC-3′		56
Nrf1	Forward: 5′-AAACCGAACACATGGCTACC-3′	168	58
	Reverse: 5′-CTGCCGTGGAGTTGAGTATG-3’		58
Tfam	Forward: 5′-TCACCTCAAGGGAAATTGAAG-3′	241	55
	Reverse: 5′-CCCAATCCCAATGACAACTC-3’		56
Long Fragment	Forward:5′-AAAATCCCCGCAAACAATGACCACCC-3’	13,400	72
	Reverse: 5′-GGCAATTAAGAGTGGGATGGAGCCAA-3’		72
Shrot Fragment	Forward: 5′-CCTCCCATTCATTATCGCCGCCCTGC-3’	235	60
	Reverse: 5′-GTCTGGGTCTCCTAGTAGGTCTGGGAA-3’		60
Bax	Forward: 5′-GCGATGAACTGGACAACAAC-3′	200	57
	Reverse: 5′-GATCAGCTCGGGCACTTTAG-3’		58
Bcl-2	Forward: 5′-CGAGTGGGATACTGGAGATGA-3’	236	58
	Reverse: 5′- GACGGTAGCGACGAGAGAAG-3’		59
Caspase-3	Forward: 5′-CCCATCACAATCTCACGGTAT-3’	195	57
	Reverse: 5′-GGACGGAAACAGAACGAACA-3’		58
Caspase-9	Forward: 5′-GCCTCTGCTTTGTCATGGAG-3’	181	56
	Reverse: 5′-AGCATGAGGTTCTCCAGCTT-3’		56
PI3K	Forward: 5′-TCACCTCCCTGATTGGCTAC-3’	220	58
	Reverse: 5′-CCACGATGGATGACAATGAA-3′		55
Akt	Forward: 5′-CGAGTCCCCACTCAACAACT-3′	231	59
	Reverse: 5′-GGTGAACCTGACCGGAAGTC-3’		60
Bad	Forward: 5′-GAGCTGACGTACAGCGTTGA-3’	153	60.39
	Forward: 5′-CCTGAGGGCTGTCCAGTAAC-3’		59.75
PARP	Forward: 5′-AAGCCTGGCACTAAGTCGAA-3’	164	59.31
	Forward: 5′-ATAGAGTAGGCGGCCTGGAT-3’		59.88
Cyc-c	Forward: 5′-GGACAGCCCCGATTTAAGTA-3’	121	57
	Forward: 5′-TCAATAGGTTTGAGGCGACAC-3’		58
GAPDH	Forward: 5′- AGGTCGGTGTGAACGGATTTG −3’	20	58
	Reverse: 5′- GGGGTCGTTGATGGCAACA-3’		58

### Measurement of mitochondrial respiratory chain complex enzymes I, II, III, IV, and V

The myocardial tissues (100 mg) were homogenized in an isolation buffer. The entire isolation process was performed at 4 °C. Mitochondrial respiratory chain complex enzymes I, II, III, IV, and V were independently assessed using the independent Assay Kit (Solarbio). Enzyme-catalyzed release were quantified by a fluorimeter at 340, 605, 550, 550, and 660 nm separately.

### Measurement of ATP

The concentration of ATP in isolated mitochondria was detected using a luciferase-based luminescence enhanced ATP assay kit (Beyotime). Isolated mitochondria (1 mg/mL protein) were incubated in respiration buffer (0.5 mL) with 2.5 mM malic acid, 2.5 mM succinate, and 2.5 mM ADP for 10 min. The ATP concentrations in the mitochondrial suspension and cell lysates were determined using a SpectraMax Paradigm Multi-Mode Microplate Reader (Molecular Devices, Sacramento, CA, USA).

### RNA extraction and cDNA synthesis

Trizol reagent (Invitrogen, Carlsbad, CA, USA) was used to isolate total genomic RNA, and the quality of isolated RNA was determined by spectrophotometry (260 nm). Reverse transcription was performed with total RNA (1 μg) and the M-MLV Reverse Transcriptase Kit (Promega A3500; Promega, Madison, WI, USA). Briefly, the total reaction volume (40 μL) was used in a Veriti 96 Well Thermal Cycler long PCR system (Applied Biosystems, Foster City, CA, USA) with the following reaction conditions: 72 °C for 3 min, 42 °C for 90 min, 70 °C for 15 min, and hold at 4 °C.

### Real-time qPCR

RT-qPCR was used to detect the copy number of specific genes at the transcriptional level using a cDNA template. PCR was performed on a Rotor-Gene Q Sequence Detection System (Qiagen, Hilden, Germany) using SYBR Premix Ex TaqII (Takara Bio, Shiga, Japan) [28]. PCR was performed in a 20 μL system (0.5 μM primers + 1 μL synthetic cDNA + 10 μL SYBR Premix Ex Taq II) under the following conditions: 95 °C for 10 min; 95 °C for 10 s, 40 cycles, 60 °C for 15 s; 72 °C for 20 s; 72 °C for 10 min. The value was calculated with GAPDH as the internal control [29]. Sequences of two pairs of PCR primers were used and are shown in Table 1.

### Protein detection

Total proteins were extracted from tissues using RIPA Lysis Buffer. Protein concentration was measured using the BCA Protein Assay Kit. An equal amount of total protein was subjected to 8–12% sodium dodecyl sulfate-polyacrylamide gel electrophoresis, and the proteins were transferred to a PVDF membrane. After blocking in skim milk solution, the membrane was incubated overnight separately with anti-GAPDH, anti-PI3K, anti-p-Akt, anti-Akt, anti-p-Bad, anti-Bad, anti-Bcl-2, anti-Bax, anti-Cyt-c, anti-caspase-9, anti-cleaved caspase-9, anti-caspase-3, anti-cleaved caspase-3, anti-PARP, anti-Drp1, anti-Mfn1, anti-Mfn2, anti-PGC-1, anti-NRF1, and anti-TFam antibodies (antibodies shown in Table 2). Then, the membrane was incubated with secondary HRP-conjugated goat anti-rabbit antibodies (Santa Cruz Biotechnology, Santa Cruz, CA, USA). The proteins were visualized using an enhanced chemiluminescence kit from Thermo Fisher Scientific (Waltham, MA, USA). Alpha View software (Cell Biosciences, Preston VIC, Australia) was used to perform the densitometric analysis.

**Table 2:** Antibodies used in the study

Antibodies	Manufacturer	Catalogue No.	Observed MW	Dilution
Anti-PI3K	Proteintech	67,071–1-1 g	110 KDa	1:10,000
Anti-p-Akt	Proteintech	66,444–1-1 g	62 KDa	1:10,000
Anti-Akt	Proteintech	10,176–2-AP	56 KDa	1:5000
Anti-p-Bad	Cell Signaling Technology	5284S	23 KDa	1:1000
Anti-Bad	Proteintech	10,435–1-AP	18 KDa	1:2500
Anti-Bcl-2	Proteintech	26,593–1-AP	26 KDa	1:2500
Anti-Bax	Proteintech	50,599–2-1 g	26 KDa	1:10,000
Anti-Caspase-3	Proteintech	19,677–1-AP	32 KDa	1:2000
Anti-cleaved-Caspase-3	Abcam	ab49822	17 KDa	1:500
Anti-Caspase-9	Proteintech	10,380–1-AP	47 KDa	1: 1000
Anti-cleaved-Caspase-9	Affinity Biosciences	AF5240	10 KDa	1: 1000
Anti-PARP1	Proteintech	13,371–1-AP	89 KDa	1:2000
Anti-Cyt-c	Proteintech	12,245–1-AP	13 KDa	1:2000
Anti-Mfn1	Proteintech	13,798–1-AP	86 KDa	1:1000
Anti-Mfn2	Proteintech	12,186–1-AP	86 KDa	1:5000
Anti-Drp1	Proteintech	10,656–1-AP	27 KDa	1:4000
Anti-PGC1a	Proteintech	66,369–1-1 g	100 KDa	1:5000
Anti-Nrf1	Proteintech	12,482–1-AP	67 KDa	1:2500
Anti-Tfam	Proteintech	22,586–1-AP	25 KDa	1:5000
Anti-GAPDH	Proteintech	60,004–1-1 g	36 KDa	1:10,000

### Statistical analysis

The SPSS statistical package (version 17.0, SPSS Inc. Chicago, IL, USA) was used for the statistical analysis. Data are expressed as mean ± standard error. One-way analysis of variance (ANOVA) was used to compare the six independent groups and the 2 × 2 comparisons between the groups. The Tukey-*t* were used to compare among the six groups. A *P*-value < 0.05 was considered significant.

## Results

### The cause of death during the experiment

Eventually, 66 rats (13 (65.00%) in the sham group, 13 (65.00%) in the IR group, 10 (50.00%) in the IR (obese) group, 10 (50.00%) in the TIIA group, 11 (55.00%) in the CsA group, and 9 (45.00%) in the TIIA+CsA group) completed this study; 54 rats died and the reasons are described in Table 3.

**Table 3:** The cause of death in the six groups rats

Cause of death	Sham	IR (non)	IR (obese)	TIIA	CsA	TIIA+CsA
Infection after injection	00	0	0	1	0	0
Massive haemorrhage	1	1	2	2	3	3
Infection after surgery	2	2	3	3	2	3
Intestinal obstruction	4	4	5	4	4	5
Number of completed cases (n (%))	13 (65.0%)	13 (65.0%)	10 (50.0%)	10 (50.0%)	11 (55.0%)	9 (45.0%)

### TIIA combined with CsA improves cardiac function, myocardial enzymes, and inflammatory factors induced by renal IR in obese rats

We assessed cardiac function (Fig. 1b), myocardial enzymes (Fig. 1c), and inflammatory factors (Fig. 1d) to assess myocardial injury and the protective effect of TIIA+CsA.

EF, FS decreased in the IR and IR (obese) groups, particularly in the IR (obese) group, compared with the sham group (*p* < 0.05)**,** which was increased by pretreatment with TIIA, CsA, and TIIA+CsA (*p* < 0.05), pretreatment with TIIA+CsA was higher than TIIA and CsA (*p* < 0.05). LVIDs and LVIDd increased in the IR and IR (obese) groups, particularly in the IR (obese) group, compared with the sham group (*p* < 0.05)**,** which was decreased by pretreatment with TIIA, CsA, and TIIA+CsA (*p* < 0.05), pretreatment with TIIA+CsA was lower than TIIA and CsA (*p* < 0.05) (Fig. 1b). The CK-MB and cTNI values increased in the IR and IR (obese) groups, particularly in the IR (obese) group, compared with the sham group (*p* < 0.05), which was decreased by pretreatment with TIIA, CsA, and TIIA+CsA (*p* < 0.05), pretreatment with TIIA+CsA was lower than TIIA and CsA (*p* < 0.05) (Fig. 1c). Serum inflammatory factor (TNF-α and IL-1β) values increased in the IR and IR (obese) groups, particularly in the IR (obese) group, compared with the sham group (*p* < 0.05), which decreased after the pretreatment with TIIA, CsA, and TIIA+CsA (*p* < 0.05), pretreatment with TIIA+CsA was lower than TIIA and CsA (*p* < 0.05) (Fig. 1d).

### TIIA combined with CsA improves the pathological structure of the myocardium induced by renal IR in obese rats

**Fig. 2:**
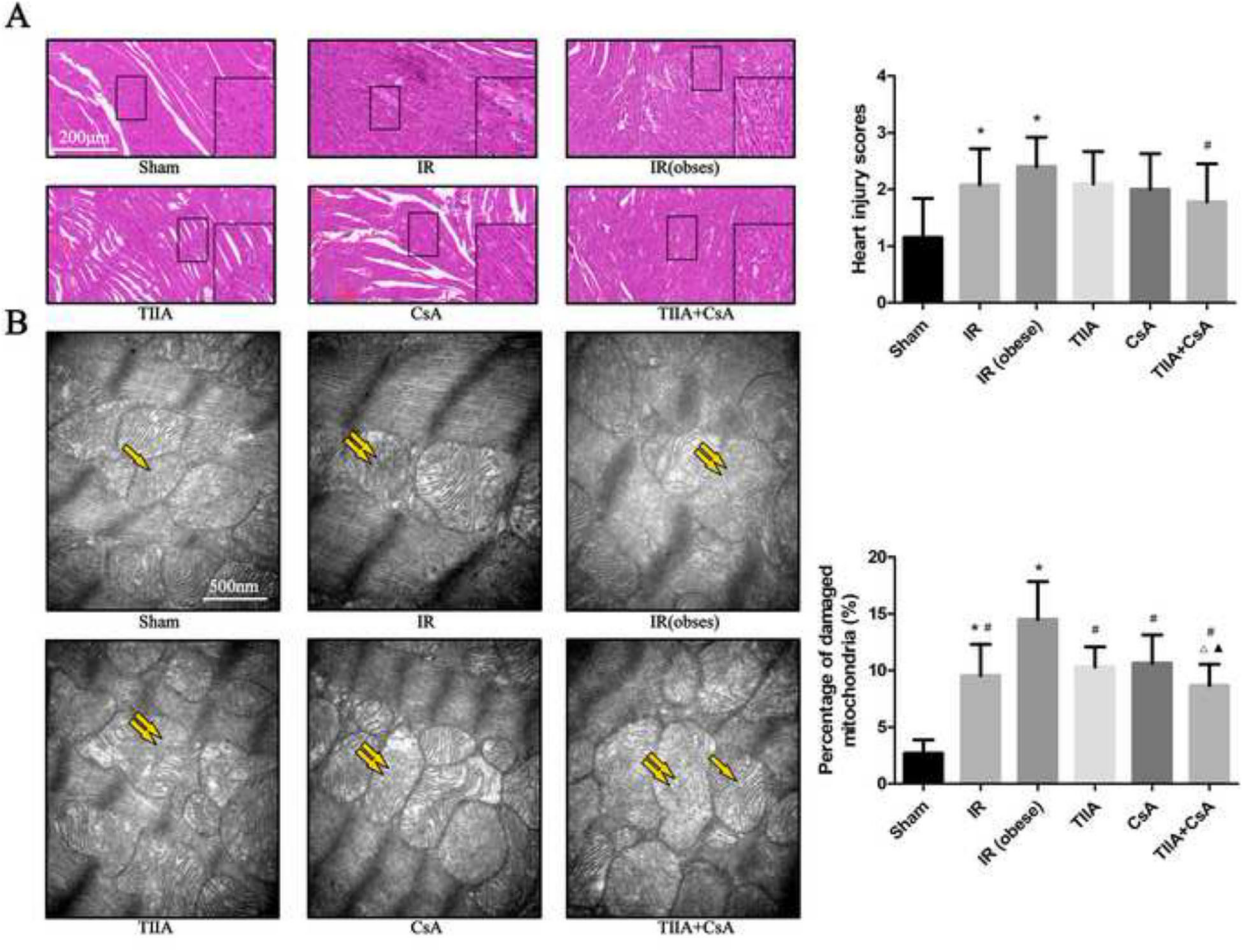
Tanshinone IIA (TIIA) + cyclosporine A (CsA) preserved myocardial architecture in renal ischemia-reperfusion (IR)-induced myocardial injury. Rats were pre-treated with TIIA alone or in combination with CsA followed by removing the right kidney and clamping of the left renal artery for 30 min and reperfusion for 24 h. Sham rats were used as control. Representative photomicrographs of myocardial histology (**a**), the scale bars represents a length of 200 μm on histology, myocardial injury scores (**a**) were evaluated under different conditions. The scale bars represents a length of 200 μm on histology. Electron microscope pictures (40000×) of rats myocardial tissue after renal ischemia-reperfusion (IR). Abnormal mitochondrial (paired yellow arrow) morphology show that mitochondrial membrane rupture or swellings, normal mitochondrial (single yellow arrow) morphology type show that mitochondrial membrane smooth and inner carinulae distinct (**b**), and percentage of damaged mitochondria (**b**). Data are shown as mean ± SD. *p < 0.05 versus sham group, ^#^*p* < 0.05 versus IR (obese) group, ^△^*p* < 0.05 versus TIIA group, ^▲^*p* < 0.05 versus CsA group. Data are shown as mean ± SD. *p < 0.05 versus sham group, ^#^*p* < 0.05 versus IR (obese) group, ^△^*p* < 0.05 versus TIIA group, ^▲^*p* < 0.05 versus CsA group

We investigated the protective effect of TIIA combined with CsA on myocardial injury after IR in obese rats by observing HE-stained myocardial tissue (Fig. 2a). The myocardial cells in the sham and TIIA+ CsA groups were similar and only had slight interstitial edema and localized necrosis. Many disordered myocardial cells combined with interstitial edema were detected in the IR and IR (obese) groups. However, pretreatment with TIIA, CsA, and TIIA+CsA alleviated the myocardial injury, particularly pretreatment with TIIA+CsA, as shown by improvements in interstitial edema, and decreased disorder of the myocardial cells. All injury changes were assessed by a histological score (Fig. 2a). The statistical results showed that the myocardial injury score in the IR and IR (obese) groups increased, particularly in the IR (obese) group, compared with the sham group (*p* < 0.05), all injury scores decreased in the TIIA, CsA, and TIIA+CsA groups, but only the TIIA+CsA group have the statistical difference (*p* < 0.05), pretreatment with TIIA+CsA was lower than TIIA and CsA but the difference is insignificant (*p* > 0.05) (Fig. 2a).

### Myocardial mitochondrial morphological changes induced by renal IR in obese rats

We evaluated mitochondrial function to assess myocardial injury and the protective effect of TIIA+CsA. We used an electron microscope to observe the changes in mitochondrial morphology (Fig. 2b).

The electron microscopic pictures (40,000×) of the myocardial tissues revealed that myocardial cells in the IR and IR (obese) groups exhibited abnormal mitochondrial morphology with swelling or membrane rupture (paired white arrows) following renal IR. The sham group exhibited normal mitochondrial morphology (single white arrow). The number of abnormal mitochondria increased in the IR and IR (obese) groups, particularly in the IR (obese) group, compared with the sham group. Pretreatment with TIIA, CsA, or TIIA+CsA improved the changes in mitochondrial morphology (Fig. 2b). Percentage of damaged mitochondria in myocardial tissue from the IR and IR (obese) groups increased compared with those in the sham group (*p* < 0.05). However, pretreatment with TIIA, CsA, or TIIA+CsA decreased percentage of damaged mitochondria (*p* < 0.05), pretreatment with TIIA+CsA was lower than TIIA and CsA (*p* < 0.05) (Fig. 2b).

### TIIA combined with CsA reduces myocardial cell apoptosis induced by renal IR in obese rats

**Fig. 3:**
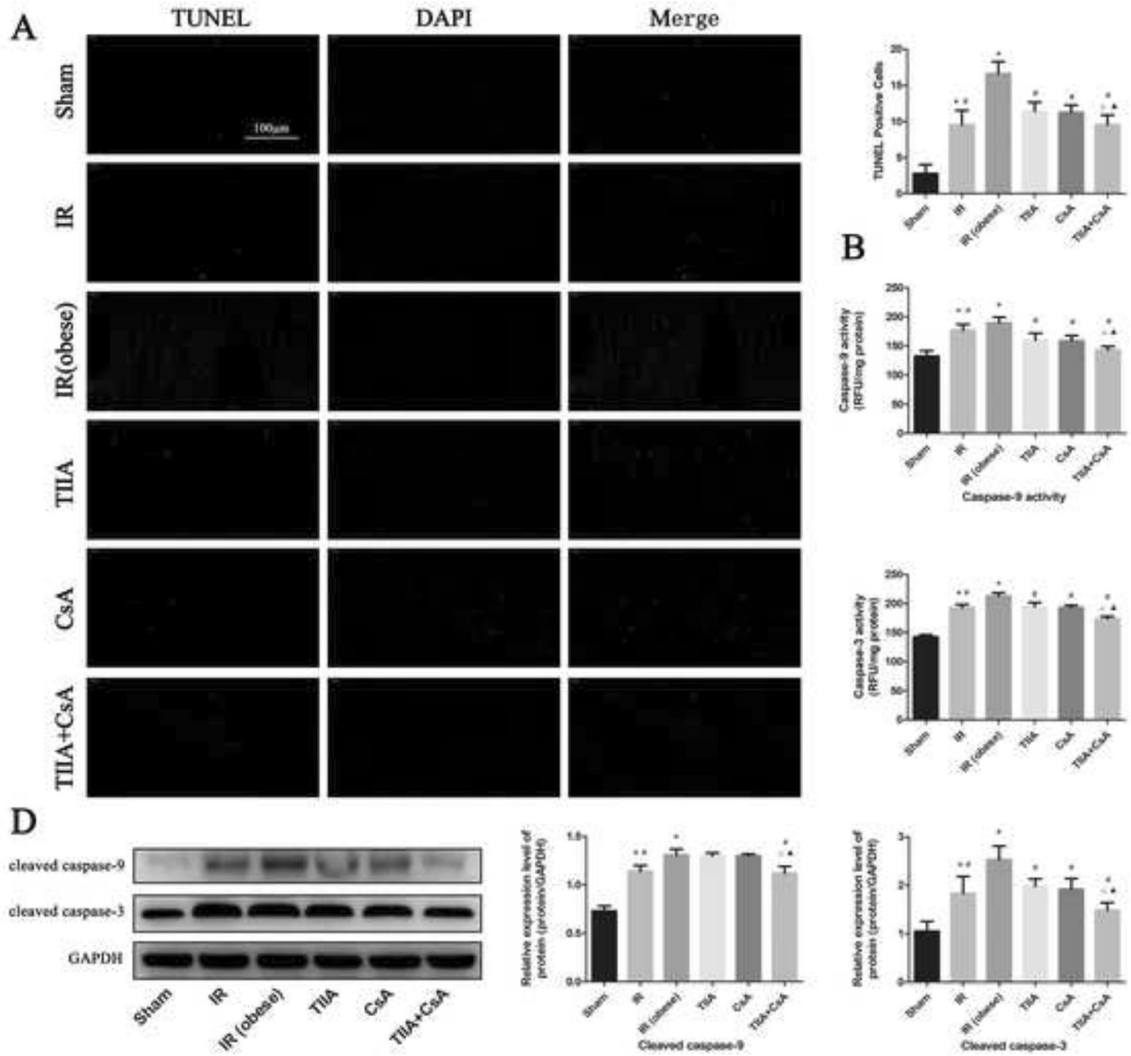
Tanshinone IIA (TIIA) + cyclosporine A (CsA) inhibited myocardial cells apoptosis after renal ischemia-reperfusion (IR). Rats were pre-treated with TIIA alone or in combination with CsA followed by removing the right kidney and clamping of the left renal artery for 30 min and reperfusion for 24 h. Sham rats were used as control. Representative apoptosis of myocardial cells (**a**), TUNEL positive cells (**a**), the scale bars represents a length of 100 μm on histology, the activity of myocardial caspase-9/3 (**b**, **c**), and the protein expression of cleaved caspase-9/3 (**d**) were evaluated under different groups. Data are shown as mean ± SD. *p < 0.05 versus sham group, ^#^*p* < 0.05 versus IR (obese) group, ^△^*p* < 0.05 versus TIIA group, ^▲^*p* < 0.05 versus CsA group

The TUNEL assay was used to investigate the protective effect of TIIA combined with CsA on myocardial cell apoptosis induced by IR in obese rats (Fig. 3a). The numbers of apoptotic cells in myocardial tissue from the IR and IR (obese) groups increased compared with those in the sham group (*p* < 0.05). However, pretreatment with TIIA, CsA, or TIIA+CsA alleviated myocardial cell apoptosis (*p* < 0.05), pretreatment with TIIA+CsA was lower than TIIA and CsA (*p* < 0.05) (Fig. 3a). Caspase-9/3 were significantly activated in the IR and IR (obese) groups than the sham group (*p* < 0.05). However, pretreatment with TIIA, CsA, or TIIA+CsA decreased caspase-9/3 activity in myocardial cells (*p* < 0.05), pretreatment with TIIA+CsA was lower than TIIA and CsA (*p* < 0.05) (Fig. 3b, c). We used western blot to detect the cleaved caspase-9/3 (Fig. 3d), the cleaved caspase-9/3 in myocardial tissue from the IR and IR (obese) groups increased compared with those in the sham group (*p* < 0.05). However, pretreatment with TIIA, CsA, or TIIA+CsA can decrease the cleaved caspase-9/3 (*p* < 0.05), pretreatment with TIIA+CsA was lower than TIIA and CsA (*p* < 0.05) (Fig. 3d).

### TIIA combined with CsA improves mitochondrial function induced by renal IR in obese rats

**Fig. 4:**
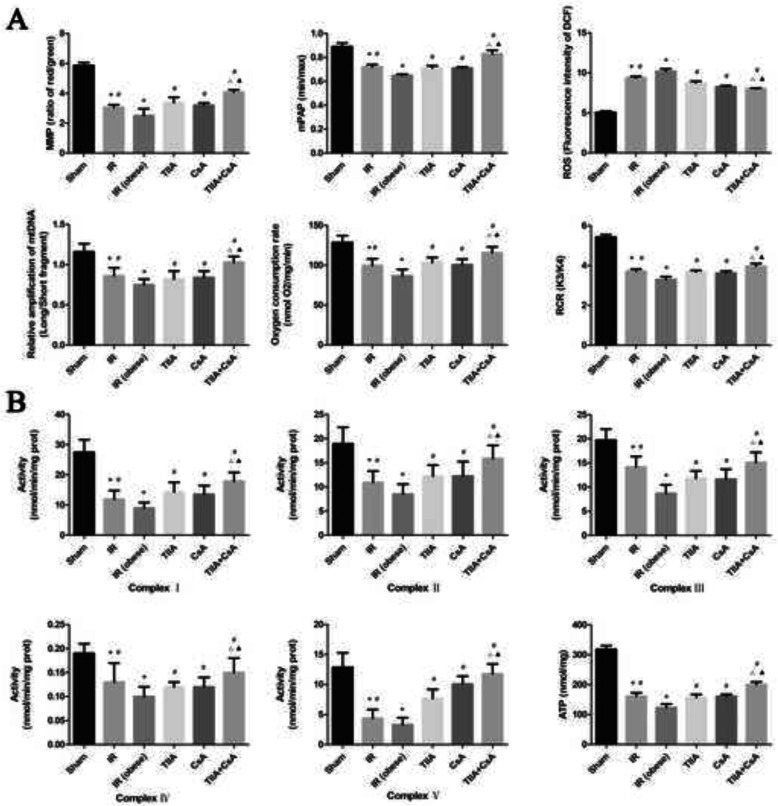
Tanshinone IIA (TIIA) + cyclosporine A (CsA) preserved myocardial mitochondrial function in renal ischemia-reperfusion (IR)-induced myocardial injury. The MMP (ratio of red/green), the opening of mPAP (%), the mitochondrial ROS, the mtDNA damage (ratio of long/short fragments), the mitochondrial RCR, mitochondrial oxygen consumption rate (**a**), the mitochondrial respiratory chain complex enzymes (I, II, III, IV, and V), and ATP (**b**) were recorded above. Rats were pre-treated with TIIA alone or in combination with CsA followed by removing the right kidney and clamping of the left renal artery for 30 min and reperfusion for 24 h. Sham rats were used as control. *p < 0.05 versus sham group, ^#^*p* < 0.05 versus IR (obese) group, ^△^*p* < 0.05 versus TIIA group, ^▲^*p* < 0.05 versus CsA group

We detected the MMP (ratio of red/green), opening of the mPTP (%), ROS, mtDNA, oxygen consumption rate, RCR, ATP, and the activity of mitochondrial respiratory chain complex enzymes (I, II, III, IV, and V) to assess myocardial mitochondrial function after renal IR and the protective effect of TIIA+CsA. Mitochondria were isolated from rat myocardial tissues. Renal IR increased the ROS level and opening of the mPTP (%), particularly in the IR (obese) group, compared with the sham group (*p* < 0.05). The ROS level and opening of the mPTP (%) were decreased by pretreatment with TIIA, CsA, or TIIA+CsA (*p* < 0.05), pretreatment with TIIA+CsA were lower than TIIA and CsA (*p* < 0.05). Renal IR decreased the mitochondrial oxygen consumption rate, RCR, and the MMP (ratio of red/green), particularly in the IR (obese) group, compared with the sham group (*p* < 0.05). Pretreatment with TIIA, CsA, and TIIA+CsA significantly increased these factors (*p* < 0.05), pretreatment with TIIA+CsA were higher than TIIA and CsA (*p* < 0.05). Real-time qPCR was used to measure the levels of mtDNA oxidative damage. The ratio of long/short fragments decreased in the IR and IR (obese) groups, particularly in the IR (obese) group, compared with the sham group (*p* < 0.05). Pretreatment with TIIA, CsA, and TIIA+CsA increased the ratio of long/short mtDNA fragments (*p* < 0.05), pretreatment with TIIA+CsA were higher than TIIA and CsA (*p* < 0.05) (Fig. 4a). Renal IR decreased the mitochondrial respiratory chain complex enzymes (I, II, III, IV, and V), and the ATP, particularly in the IR (obese) group, compared with the sham group (*p* < 0.05). Pretreatment with TIIA, CsA, and TIIA+CsA significantly increased these enzymes (*p* < 0.05), pretreatment with TIIA+CsA were higher than TIIA and CsA (*p* < 0.05) (Fig. 4b).

### TIIA combined with CsA improves mitochondrial biogenesis and the dynamics induced by renal IR in obese rats

**Fig. 5:**
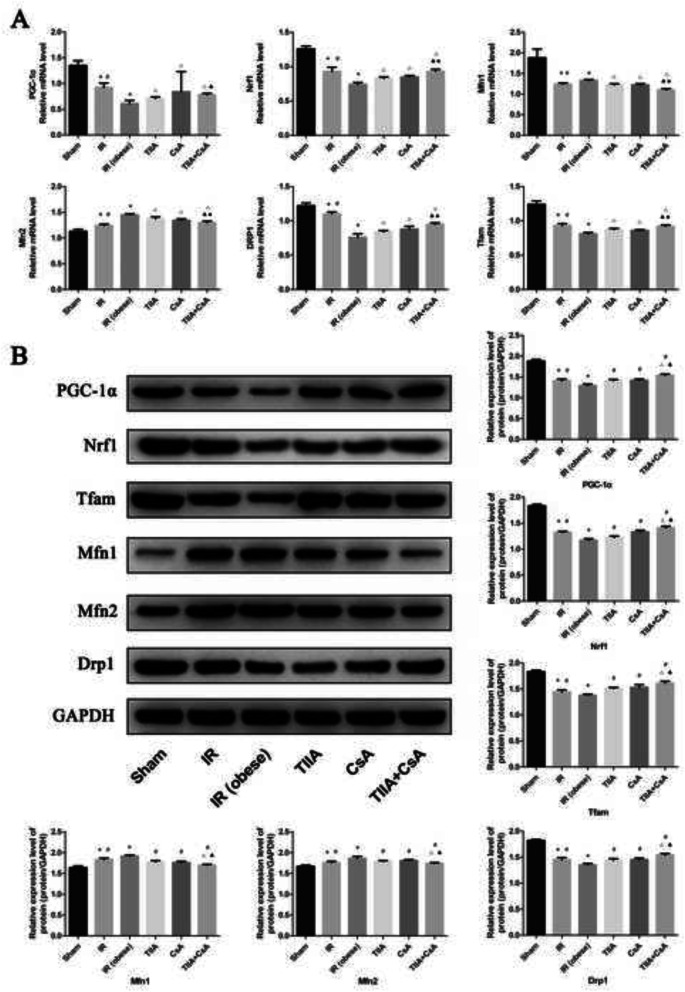
Tanshinone IIA (TIIA) + cyclosporine A (CsA) preserved mitochondrial biogenesis and dynamics in renal renal ischemia-reperfusion (IR)-induced myocardial injury. The expression of PGC-1α, Nrf1, and Tfam in mRNA (**a**) and protein (**b**) level. The expression of Mfn1, Mfn2, and Drp1 in mRNA and protein level level. *p < 0.05 versus sham group, ^#^*p* < 0.05 versus IR (obese) group, ^△^*p* < 0.05 versus TIIA group, ^▲^*p* < 0.05 versus CsA group

We used real-time qPCR and western blot to detect mitochondrial biogenesis and dynamics. We chose PPARγ coactivator-1-α (PGC-1α), nucleo respiratory factor1 (Nrf1), and transcription factor A of mitochondrial (Tfam) to represent mitochondrial biogenesis, and dynamin-related protein 1 (Drp1; fission) and mitofusins (Mfns; Mfn1 [fusion], Mfn2 [fusion]), and Drp1 to represent mitochondrial dynamics (fusion and fission processes). Our results show that PGC-1α, Nrf1, Tfam, and Drp1 mRNA levels decreased in the IR and IR (obese) groups, particularly in the IR (obese) group, compared with the sham group (*p* < 0.05). Pretreatment with TIIA, CsA, and TIIA+CsA increased these mRNA levels (*p* < 0.05), pretreatment with TIIA+CsA were higher than TIIA and CsA (*p* < 0.05). Mfn1 and Mfn2 mRNA expression increased in the IR and IR (obese) groups, particularly in the IR (obese) group, compared with the sham group (*p* < 0.05). The mRNA levels of these factors decreased after pretreatment with TIIA, CsA, and TIIA+CsA (*p* < 0.05), pretreatment with TIIA+CsA were lower than TIIA and CsA (*p* < 0.05) (Fig. 5a). The western blot of the biogenesis and dynamics factors showed consistent results (Fig. 5b).

### TIIA combined with CsA modulates the PI3K/Akt/bad pathway

**Fig. 6:**
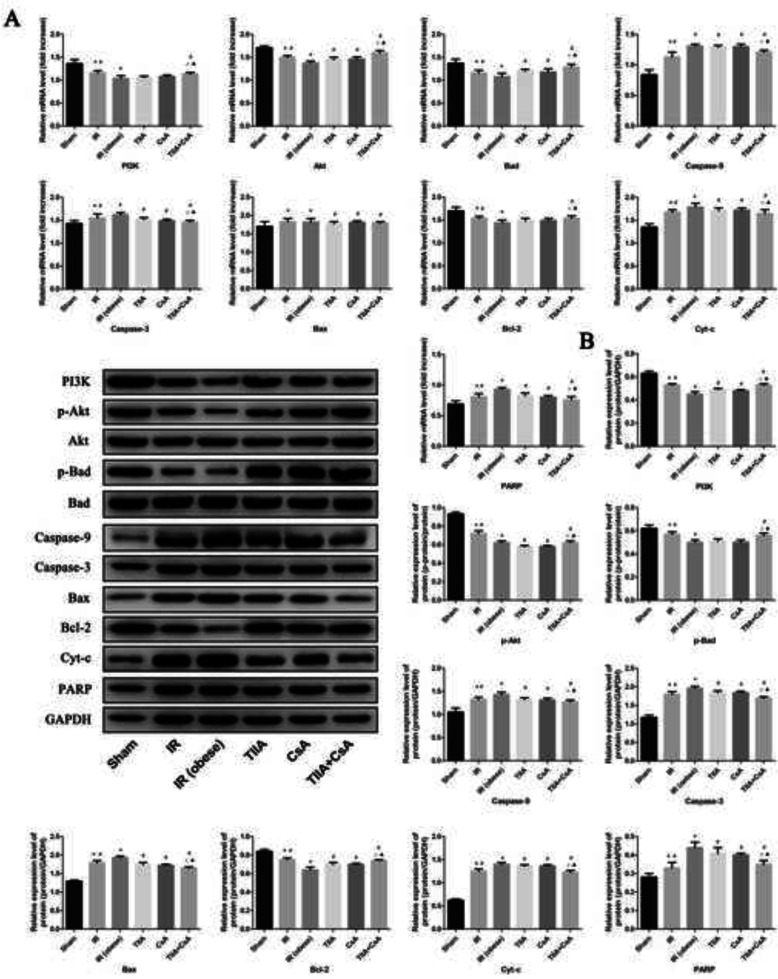
Tanshinone IIA (TIIA) + cyclosporine A (CsA) modulated myocardial PI3K/Akt/Bad pathway. The expression of PI3K, p-Akt, Akt, p-Bad, Bad, Bax, Bcl-2, Cyt-c, caspase-9, caspase-3, and PARP in mRNA (**a**) and protein (**b**) level. *p < 0.05 versus sham group, ^#^*p* < 0.05 versus IR (obese) group, ^△^*p* < 0.05 versus TIIA group, ^▲^*p* < 0.05 versus CsA group

We used real-time qPCR and western blot to detect the target proteins in the PI3K/Akt/Bad pathway in mRNA and protein levels. Renal IR increased the mRNA level expression of Bax, Cyt-c, caspase-9, caspase-3, and PARP significantly, particularly in the IR (obese) group, compared with the sham group (*p* < 0.05). The mRNA level expression of these factors decreased after pretreatment with TIIA, CsA, and TIIA+CsA (*p* < 0.05), pretreatment with TIIA+CsA were lower than TIIA and CsA (*p* < 0.05). Renal IR decreased the mRNA level expression of PI3K, Bad, Akt, and Bcl-2 significantly, particularly in the IR (obese) group, compared with the sham group (*p* < 0.05). The mRNA level expression of these factors increased after pretreatment with TIIA, CsA, and TIIA+CsA (*p* < 0.05), pretreatment with TIIA+CsA were lower than TIIA and CsA (*p* < 0.05) (Fig. 6a). The western blot of the biogenesis and dynamics factors showed consistent results (Fig. 6b). TIIA, CsA, and TIIA+CsA induced Akt and Bad phosphorylation (enhanced p-Bad/Bad and p-Akt/Akt) (Fig. 6b).

## Discussion

The main cause of AKI in critically patients is renal ischemia-reperfusion injury (IRI). Continuous renal replacement therapy is the main AKI treatment for critically ill patients, however, it is not sufficient to reduce mortality, multiple organ dysfunction syndrome, and interorgan crosstalk induced by extrarenal complications, which are causes of death from AKI [30]. Renal IRI increases the inflammatory response and cytokine levels, which damage remote organs, such as the heart and lungs [30]. Hyperlipidemia is a risk factor for cardiovascular disease, and it can increase damage by increasing inflammation and ROS [31]. In our study, we prepared the obesity model by feeding the HFD. Renal IRI stimulates a series of complex changes in cells that induce necrosis and apoptosis in cells. Oxidative stress increases inflammation and ROS induce the release of pro-inflammatory mediators during the reperfusion phase. These factors play an important role in the pathophysiological course of renal IR. Several antioxidants and anti-inflammatory agents play effective roles reducing the degree of injury. These antioxidants increase the survivability of the kidney to IR injury [32].

AKI often combines with AMI in critically ill patients. Despite that there is a definite relationship between the kidney and the heart, the specific mechanism of the pathophysiological change between the kidney and the heart is unknown. This phenomenon has become controversial because of the confusing results. Despite patient use of dialysis, mortality (50%) is still very high [33], so a new method must be identified to decrease mortality from AKI. In our study, we prepared the IRI renal model in obese rats and discovered that renal IRI decreased cardiac function, particularly in obese rats (*p* < 0.05), and that TIIA, CsA, and TIIA+CsA improved cardiac function (*p* < 0.05), pretreatment with TIIA+CsA was better than TIIA and CsA (*p* < 0.05) (Fig. 1b). Additionally, renal IRI increased myocardial enzyme (CK-MB and cTNI) levels, particularly in obese rats (*p* < 0.05). In contrast, TIIA, CsA, and TIIA+CsA decreased myocardial enzyme (*p* < 0.05), pretreatment with TIIA+CsA was lower than TIIA and CsA (*p* < 0.05) (Fig. 1c). Taken together, renal IRI induced myocardial injury (decreased cardiac function and increased myocardial enzymes), whereas TIIA, CsA, and TIIA+CsA improved myocardial injury, particularly pretreatment with TIIA+CsA.

Renal IRI is involved in the adaptive and innate immune responses and releases cytokines in the kidney [34]. Once this process starts, soluble mediators and remote organs, such as the lung and heart, become damaged through organ crosstalk. The kidney-heart interaction includes several inflammatory mediators in the pathophysiological process of cardiorenal syndrome [35], and the elevated levels of inflammatory cytokines adversely affect myocardial function.

Increased levels of TNF-α and IL-6 are related with the development of congestive heart failure and mortality in patients with congestive heart failure [36]. Several pathways, such as activation of inflammatory transcription factors and the induction of inflammatory cytokines and genes, contribute to heart damage following renal IRI. In our study, renal IRI induced AMI. We used enzyme-linked immunosorbent assays to detect TNF-α and IL-1β, and our results show that renal IRI increased serum levels of TNF-α and IL-1β, particularly in the IR (obese) group, compared with the sham group (*p* < 0.05), which decreased after the pretreatment with TIIA, CsA, and TIIA+CsA (*p* < 0.05), pretreatment with TIIA+CsA was lower than TIIA and CsA (*p* < 0.05) (Fig. 1d). Systemic inflammation regulates cardiac tissue structure, as distinct sections of heart tissues have different collagen content [7].

HE staining of rat myocardial tissue revealed that renal IRI induced a disordered structure, edema, and localized necrosis in myocardial cells, particularly in the IR (obese) group, combined with interstitial edema. However, pretreatment with TIIA, CsA, and TIIA+CsA alleviated the myocardial injury, particularly pretreatment with TIIA+CsA, as shown by improvements in interstitial edema and decreases in the number of disordered myocardial cells (Fig. 2a). Structural damage to myocardial tissues also decreased cardiac function (Fig. 1b). The statistical results showed that the myocardial injury score in the IR and IR (obese) groups increased compared with that in the sham group (*p* < 0.05). The score in the IR (obese) group was higher than that in the IR group (*p* < 0.05). All injury scores decreased in the TIIA, CsA, and TIIA+CsA groups, but only the TIIA+CsA group have the statistical difference (*p* < 0.05), pretreatment with TIIA+CsA was lower than TIIA and CsA but the difference is insignificant (*p* > 0.05) (Fig. 2a). At the same time, the TUNEL assay showed that renal IRI induced myocardial cell apoptosis (Fig. 3a), whereas TIIA+ CsA decreased the number of apoptotic cells (Fig. 3a). Caspases are major executors of apoptosis related to the final stages of apoptosis. Activation of effector caspase-9/3 is an important indicator of apoptosis, so caspase-9/3 activity was measured in the present study. Caspase-9/3 was significantly activated in the IR and IR (obese) groups compared to the sham group, particularly in the IR (obese) group (*p* < 0.05). However, TIIA, CsA, or TIIA+CsA decreased caspase-9/3 activity in myocardial cells (*p* < 0.05), pretreatment with TIIA+CsA was lower than TIIA and CsA (*p* < 0.05) (Fig. 3b, c). These results are similar to those of Juanjuan Li et al [37] in an acute lung injury model induced by renal IRI. As the active form of caspase-3, cleaved caspase-3 can induce apoptosis, IR can increase the cleaved caspase-9/3, However, pretreatment with TIIA, CsA, or TIIA+CsA can decrease the cleaved caspase-9/3, pretreatment with TIIA+CsA was better than TIIA and CsA (*p* < 0.05) (*p* < 0.05) (Fig. 3d).

According to previous studies, some anti-inflammatory agents have been discovered, including α-melanocyte stimulating hormone, anti-apoptotic agents, and IL-6 inhibitors [37, 38]. A previous study reported that renal IR induces dysfunction in lung mitochondria and dexmedetomidine attenuates lung inflammation, apoptosis, and the MMP [37]. However, until now, no study has investigated myocardial mitochondrial dysfunction induced by renal IR, and no anti-mitochondrial dysfunction agents have been discovered to protect against AMI induced by renal IR in obese rats. Using only the MMP to evaluate the mitochondrial function is too circumscribed, so more methods must be used to evaluate mitochondrial function. We chose the MMP, mitochondrial ATP, opening of the mPTP, mitochondrial ROS, mtDNA, and mitochondrial biogenesis/dynamics to comprehensively evaluate mitochondrial function.

As a universal medicinal herb, it has been demonstrated that S. *miltiorrhiza* Bge eliminates toxic substances from the blood, accelerates fibrinolytic enzyme activity and thrombolysis, reduced blood viscosity, and protects the cardiovascular system and blood vessels [38]. TIIA is the main active ingredient in *S. miltiorrhiza* Bge. Pretreatment with TIIA relieves renal injury induced by IR by downregulating the expression of myeloperoxidase (MPO), caspase-3, and inflammation [38]. TIIA decreases the generation of ROS and release of Cyt-c, reduces the frequency of apoptosis, inactivates caspase-3, and inhibits opening of the mPTP [39]. Because CsA inhibited opening of the mPTP, it has been implicated as a treatment target at the beginning of reperfusion [39]. It is now widely believed that mitochondrial dysfunction (particularly opening of the mPTP) plays an important role aggravating injury following renal IRI [40]. Opening of the mPTP decreased the membrane potential and mitochondria swelling, which inhibited oxidative phosphorylation. Opening of the mPTP occurs through binding of the CyP-D protein to the mitochondrial inner membrane. Previous studies have shown that CsA protects against IRI through CyP binding, independently of the anti-calcineurin properties; thus, inhibiting opening of the mPTP [41]. However, no study has explored whether CsA inhibits opening of the mPTP to inhibit the myocardial mitochondrial dysfunction induced by renal IRI. Mitochondrial function is the pathophysiological bridge between kidney-heart interactions during renal IRI. We hypothesized that TIIA combined with CsA would play a protective role in ALI by improving mitochondrial function.

Mitochondria are the energy center of the cell, supplying more than 95% of the ATP for metabolism [42]. Thus, mitochondria are a logical point to define the pathophysiological processes and therapeutic targets in various metabolic diseases. To explore the relationship between the heart and kidney, we established the renal IR rat model by removing the right kidney and clamping the left renal artery for 30 min followed by reperfusion for 24 h. We then pre-treated the rats with TIIA combined with CsA to determine mitochondrial function. As shown in Fig. 4, renal IRI induced AMI, and the electron microscopic photographs (40,000×) of myocardial tissue revealed that renal IRI induced abnormal mitochondrial morphology and increased the percentage of damaged mitochondria, such as mitochondrial swelling and membrane rupture, particularly in the obese model rats. TIIA, CsA, or TIIA+CsA alleviated the mitochondrial injury, particularly pretreatment with TIIA+CsA (Fig. 2b). Oxygen consumption rate and RCR decreased in the IR and IR (obese) groups, particularly in the IR (obese) group, compared with the sham group (*p* < 0.05). Pretreatment with TIIA, CsA, and TIIA+CsA alleviated the decrease in the oxygen consumption rate and RCR, pretreatment with TIIA+CsA was lower than TIIA and CsA (*p* < 0.05) (Fig. 4a). Renal IR significantly increased the ROS level and opening of the mPAP (%) (*p* < 0.05). TIIA, CsA, and TIIA+CsA significantly decreased the ROS level and opening of the mPAP (%), pretreatment with TIIA+CsA was lower than TIIA and CsA (*p* < 0.05) (Fig. 4a). Renal IR decreased the MMP (ratio of red/green) significantly (*p* < 0.05), whereas TIIA, CsA, and TIIA+CsA increased the mitochondrial MMP (ratio of red/green) significantly, pretreatment with TIIA+CsA was higher than TIIA and CsA (*p* < 0.05) (Fig. 4a). These results are similar to those of Luan G et al [24].

The mtDNA copy number in each mitochondrion is stable, so the total copy number of mtDNA can be used to estimate the quantity of mitochondria [43]. Although the mtDNA repair mechanism is unknown, mtDNA is near the respiratory chain, so it is more vulnerable when exposed to oxidative reactions, compared to nuclear DNA. In our study, we observed the damaging effect of renal IR on mtDNA and the protective effect of TIIA+CsA, using the ratio of long and short fragments to assess the degree of damage. Our results show that the ratio of long/short fragments decreased in the IR and IR (obese) group, particularly in the IR (obese) group, compared with the sham group (*p* < 0.05), which increased after pretreatment with TIIA, CsA, and TIIA+CsA, pretreatment with TIIA+CsA was higher than TIIA and CsA (*p* < 0.05) (*p* < 0.05) (Fig. 4a). As mtDNA was damaged by renal IR, synthesis of the complex proteins in the mitochondrial electron transport chain would be inhibited, resulting in obstruction of electron transport.

Respiration is one of the most vital and basic features of living organisms. In mammals, respiration is accomplished by respiratory chain complexes located on the mitochondrial inner membrane to transfer electrons and establish the proton gradient for complex V to synthesize ATP [44]. As shown in Fig. 4b, Renal IR decreased the mitochondrial respiratory chain complex enzymes (I, II, III, IV, and V), and the ATP, particularly in the IR (obese) group. Pretreatment with TIIA, CsA, and TIIA+CsA significantly increased these enzymes, pretreatment with TIIA+CsA was higher than TIIA and CsA (*p* < 0.05).

As an important adaptation to exposure to chronic energy deprivation, mitochondrial biogenesis is modulated by many complex elements, such as Tfam and Nrf1. Nrf1 fosters transcription of nuclei-encoded mitochondrial protein expression, including factors involved in the respiratory complexes and oxidative phosphorylation. Tfam increases gene transcription and DNA replication in mitochondria by directly binding to the mitochondrial genome. PGC-1α is an important transcriptional co-activator that modulates key factors, including Nrf1 and Tfam, which increase mitochondrial biogenesis [45]. Mitochondrial biogenesis becomes chaotic when expression of these gene changes. As shown in Fig. 5, renal IR reduced the expression of PGC-1α, Nrf1, and Tfam. TIIA+CsA markedly increased the levels of PGC-1α, Nrf1, and Tfam. After the TIIA, CsA, or TIIA+CsA treatment, increased mitochondrial oxygen consumption rate, a sufficient energy supply (ATP), decreased ROS, and improved mitochondria biogenesis factors acted synergistically to improve the intracellular energy supply shortage and the effect of TIIA+CsA was better than TIIA and CsA. Harmful stimuli, including oxidative stress, aging, and energy limitations damage mitochondria, which become fused to lysosomes and degraded. Autophagic abnormalities in mitochondria increase the number of damaged mitochondria [24].

Mitochondria normally undergo a dynamic process of fusion and fission. This dynamic process is important to maintain constant changes in size, shape, and network of mitochondria. Mitochondria are under the control of regulatory proteins, including Drp1 and Mfns [46]. Our results (Fig. 5) show that the expression of Mfns (Mfn1 and Mfn2) and Drp1 changed after renal IR, showing a chaotic balance of fission-fusion in mitochondria. Mfns are believed to have an important role in mitochondrial fusion, and Drp1 may have an important role in mitochondrial fission. We observed increases in Mfn1 and Mfn2, and a decrease in Drp1 in myocardial tissues after renal IR.

The PI3K/Akt/Bad signaling pathway plays an important role inhibiting apoptosis mediated by mitochondria [15]. This pathway can regulate the expression of downstream apoptosis proteins (Bax and Bcl-2), and it can regulate the cell migration, growth, angiogenesi, invasion [47]. Activiting the PI3K-AKT signalling pathway can inhibit apoptosis through dephosphorylating phosphatidylinositol triphosphate to phosphatidylinositol diphosphate [48]. Because of the close relationship between them, we researched the effect of PI3K in this study. PI3K is a phosphatidylinositol kinase with activities as a serine/threonine-specific protein kinase and a phosphatidylinositol kinase [49]. After activation, phosphatidylinositol family members are phosphorylated on the cell membrane and the downstream signal molecule Akt is recruited and activated. Activated Akt phosphorylates the Ser136/Ser112 residues of the Bad protein [50]. Phosphorylated Bad separates from the apoptosis-promoting complex and forms the 14–3-3 protein complex, leading to inactivation of its apoptosis-promoting function, which inhibits apoptosis [51]. Our results show that TIIA, CsA, or TIIA+CsA effectively regulated the expression of apoptotic PI3K/Akt/Bad pathway-related proteins, and TIIA, CsA, or TIIA+CsA enhanced PI3K and p-Akt expression but downregulated expression of Cyt-c, caspase-9/3, and PARP, the effect of TIIA+CsA was better than TIIA and CsA (Fig. 6). These results show that TIIA+CsA adjusted mitochondrial function and inhibited apoptosis of myocardial cells induced by renal IRI by activating the PI3K/Akt/Bad pathway.

In our study, we used isolated mitochondria to show that renal IR promotes the production of ROS, which damages mtDNA, mitochondrial respiratory function, biogenesis, and dynamic function. Swollen mitochondria were induced by opening of the mPTP after renal IR. Opening of the mPTP induced back flow of protons from the mitochondrial membrane space into the matrix, thereby reducing ATP synthesis and MMP, leading to metabolic abnormalities. A reduction of MMP and ATP synthesis and an increase of Cyt-c were induced by opening of the mPTP, which led to back flow of protons from the mitochondrial membrane space to the matrix to induce metabolic abnormalities and eventually leading to myocardial cell apoptosis. The TIIA+CsA pre-treatment inhibited apoptosis by modulating mitochondrial function through the PI3K/Akt/Bad pathway in obese rats. But in our study, considering of the combination therapy, we did not use the PI3K/Akt/Bad pathway inhibitor, so the mechanism of drug intervention can not be fully revealed,in the future, we will use the LY294002 (PI3K inhibitor) in the in vitro cell experiment in order to fully reveal the mechanism of its intervention in the future.

## Conclusions

Renal IR can induce the mitochondrial dysfunction and apoptosis (obesity increases the severity) in myocardial cell, TIIA combined with CsA can attenuated myocardial cell apoptosis by modulating mitochondrial function through the PI3K/Akt/Bad pathway in obese rats.

## Data Availability

The datasets used and/or analysed during the current study are available from the corresponding author on reasonable request.

## Abbreviations

IR: ischemia-reperfusion
AKI: acute kidney injury
AMI: acute myocardial injury
CsA: cyclosporine A
TIIA: Tanshinone IIA
HE: hematoxylin-eosin
TUNEL: terminal deoxynucleotidyl transferase-mediated dUTP nick end-labeling
MMP: mitochondrial membrane potential
ATP: adenosine triphosphate
ROS: reactive oxygen species
RCR: respiration controlling rate
mPTP: mitochondrial permeability transition pore
CVD: cardiovascular diseases
CyP-D: cyclophilin D
DMSO: dimethylsulfoxide
HFD: high-fat diet
SD: Sprague-Dawley
PBS: phosphate buffer saline
CK-MB: creatine kinase isoenzymes-muscle/brain
cTNI: cardiac troponin I
IL-1β: interleukin-1β
TNF-α: tumor nerosis factors-α
TM: time-motion
LVIDs: left ventricular end-systolic internal diameter
LVIDd: left ventricular end-diastolic internal diameter
EF: ejection fraction
FS: fractional shortening
BCA: bicinchoninic acid
OD: optical density
DAPI: 4′,6-diamidino-2-phenylindole
EDTA: ethylene diamine tetraacetic acid
DCFH-DA: 2′,7′-Dichlorodihydrofluorescein diacetate
RT-qPCR: Real-time quantitative Polymerase Chain Reaction
ANOVA: one-way analysis of variance
SDS-PAGE: sodium dodecyl sulfate-polyacrylamide gel electrophoresis
IRI: ischemia-reperfusion injury
ELISA: euzymelinked immunosorbent assay
PI3K: Phosphoinositide-3 kinase
Cyt-c: cytochrome C
PARP: poly ADP-ribose polymerase
Drp1: dynamin-related protein 1
Mfns: Mitofusins
PGC-1α: PPARγ coactivator-1-α
Nrf1: Nucleo respiratory factor1
Tfam: Transcription factor A of mitochondrial
